# Evaluation Models for Undergraduate Nursing Clinical Skills: A Scoping Review Protocol

**DOI:** 10.2196/79805

**Published:** 2026-03-13

**Authors:** Kateryna Metersky, Tommy Lin, Tina Lam, Areej Al-Hamad, Daria Romaniuk

**Affiliations:** 1 Daphne Cockwell School of Nursing, Toronto Metropolitan University, 288 Church St, Toronto, ON, M5B 1Z5, Canada, 1 4169795000 ext 544906

**Keywords:** clinical skills assessment, nursing, nursing curricula, nursing student, skills testing

## Abstract

**Background:**

Undergraduate nursing students are expected to perform a high-stakes clinical skills test, which ultimately determines their ability to engage in clinical practice. With intake number of students growing nationally, clinical instructors are modifying these skills tests to be shorter in duration as an attempt to meet scheduled class times, severely decreasing the assessments’ accuracy and increasing student stress.

**Objective:**

The objectives of this review are to examine best practices of evaluation structures or models for undergraduate nursing students participating in clinical skills-based assessments as part of their program curriculum to explore alternative, best-practice methods for evaluating key clinical skills. The goal is to use what is known in the literature to help inform the development of a new evidence-based clinical skills evaluation model that is adaptive to student population growth, enhances student learning and faculty teaching experiences, and ensures safe patient care.

**Methods:**

This scoping review will consider studies involving nursing students enrolled in an undergraduate nursing degree program who undergo clinical skills testing as part of their degree fulfillment. Original articles published in English from January 1, 2015, to present day will be included. This review will follow the Joanna Briggs Institute methodology for scoping reviews, as well as the Preferred Reporting Items for Systematic Reviews and Meta-Analyses extension for Scoping Reviews (PRISMA-ScR) guidelines. The literature search will use the following databases: SCOPUS, CINAHL, Medline, PsycInfo, ProQuest Central, and ProQuest Dissertations and Theses to identify relevant sources. Moreover, nonempirical research, such as editorials, opinion papers, gray literature, and reviews, will be included. Data from professional nursing organizations, including the College of Nurses of Ontario (CNO), the Registered Nurses’ Association of Ontario (RNAO), and the Canadian Association of Schools of Nursing (CASN), will be considered. Two independent reviewers will conduct a screening of article titles and abstracts, followed by full-text reviews. Together, they will determine which articles will proceed to the data extraction stage. If discrepancies arise, a consensus discussion will take place between the two reviewers with assistance from a third reviewer.

**Results:**

The project was funded in June 2025 (Figure 1). The scoping review is scheduled to begin in July 2025, with the literature search and study selection processes planned through Fall 2025. Results are expected to be submitted for publication in Winter 2026.

**Conclusions:**

The results of this review will summarize the current breadth of knowledge on clinical skills testing and best-practice methods used for clinical skill evaluation among undergraduate nursing students. Any gaps in the literature will be identified as these can be used to guide future research within this area of nursing education.

## Introduction

Standardized skills testing has been used as a method of evaluation for undergraduate students in nursing programs across Canadian schools to assess and measure student competence in executing essential nursing skills. In such programs, students are prohibited from entering clinical practice or performing such skills in a clinical setting until they have successfully demonstrated competence in performing the skills in the laboratory setting. The assessment typically or most often consists of an individual one-on-one evaluation of a sequential demonstration of a psychomotor skill, such as wound care, Foley catheterization, intramuscular injection as examples, to be carried out within a designated timeframe. Such skills testing is viewed as a high-stakes assessment, meaning that students who fail typically must undergo remediation until they are deemed successful [1].

This model of testing, in which students would face significant consequences if their performance was not evaluated as successful, is quite stressful for students. Such consequences can include inability to move on to in-person clinical practice or having to remain at a certain level in the program until they are successful. Such consequences may make their educational experience stressful and give rise to increased preparation and test-taking anxiety [1,2]. Such an impact on students could potentially lead to poor performance through increased mistake-making during testing despite the student having the requisite knowledge [3]. Nursing students have often expressed that skill pass-offs invoked negative responses and emotions related to this one-time high-stakes assessment, such as fear and being in a high pressure situation, resulting in decreased confidence when performing the required skills [4].

The supply of Canadian nurses has not kept pace with the country’s ever-increasing demand for health care services, with forecasting models documenting “a shortage of 60,000 nurses nationwide by 2022 and more than 117,000 by 2030” [5]. Due to current health human resource shortages experienced in acute care settings nationally, both provincial and territorial governments have put forth a call to expand the health care workforce and consequently, increase the number of seats for nursing students across postsecondary institutions. This continual increase in student enrollment numbers without any increase in physical space, nursing instructors, nursing education resources, or instructional hours has already posed significant challenges for the traditional skills testing model [6]. Class size, time, faculty resistance, and workload concerns remain considerable barriers for nursing educators to effectively teach and evaluate clinical competence [7]. Faculty find it increasingly challenging to adequately and fairly assess their students under such constraints [8]. As a result, instructors have been prompted to come up with creative solutions to ensure all students can get tested within the same amount of class time, despite the significant increase in student numbers. Thus, instructors have started to modify the traditional assessment through either decreasing the time students have to demonstrate the entire skill or asking students to come prepared to perform the entire skill, but assigning only a small component of the skill to demonstrate during the testing.

Altering the skills testing model in this manner may result in an unreliable evaluation of students’ competence and capability to perform the requisite skill on a real patient in the clinical setting, decreasing the integrity and validity of this assessment. Presently, these adjustments are seldom aligned with the best practices for effective clinical skills evaluation of students. These modifications are a ‘cookie-cutter’ approach that can lead to issues such as diminishing the integrity of the evaluation process, heightening student anxiety and instructor fatigue, and necessitating both parties to exceed beyond their scheduled class hours to ensure every student is tested prior to entering the clinical setting at a timely point in the semester. Faculty members conclude that more uniformity is needed in how learning outcomes are interpreted and how students are being assessed, advocating for an evaluation process that is more consistent and reliable with clear expectations for both students and educators [9]. There is a strong need to readdress undergraduate nursing curricula regarding clinical competence in order to prepare nursing graduates for the everchanging profession [10]. Failure to accurately assess students and a lack of appropriate educational support as a means for clinical skills acquisition may constitute a threat to patient safety down the line [11]. Therefore, it is necessary for educators to determine students’ learning needs and thoroughly identify skill and knowledge gaps that could hinder the development of clinical competence to adequately meet professional nursing care standards [9-11].

Nursing curricula must be designed to equip graduates with the knowledge and skills necessary to meet present and emerging health care demands. There is a need to strengthen students’ competence and confidence by integrating new technologies and innovative teaching approaches into traditional methods [12,13]. It is important for educators to emphasize the learning process as a whole rather than focusing solely on the outcomes by thoughtfully pacing and adjusting student support throughout the nursing program. This foundational pedagogy continues to play an important role in contemporary education [14]. Evidence-based practices within educational approaches can help improve the clinical outcome through increased knowledge, skill acquisition, and competency, as well as improve the beliefs, attitudes and behaviors of undergraduate students [15].

This scoping review aims to explore alternative, best-practice methods for evaluating key clinical skills in nursing students, with the goal of informing the development of a new evidence-based evaluation model. The model could better accommodate student population growth, enhance the overall learning experience for students as well as teaching experience for instructors, and guarantee safe patient care in clinical settings. The review addresses the central question: What is known in the literature about how key nursing skills are evaluated in undergraduate nursing students within Canada? Analyzing relevant scholarly and gray literature will reveal the depth of knowledge within current literature regarding best practices for the assessment of nursing clinical skills in a laboratory environment and generate key themes that will ultimately guide the creation of a new evaluation model.

## Methods

### Study Design

This scoping review will apply the Joanna Briggs Institute (JBI) methodology due to its iterative structure and comprehensive framework for scoping reviews [16]. The selection of sources, data extraction, and presentation of findings will be guided by the Preferred Reporting Items for Systematic Reviews and Meta-Analyses Extension for Scoping Reviews (PRISMA-ScR) for its structured and rigorous review process [17]. The protocol was developed using Preferred Reporting Items for Systematic Reviews and Meta-Analysis Protocols extension (PRISMA-P) [18].

### Inclusion Criteria

The Population, Concept, and Context framework will be used to structure the scope and inclusion criteria of the scoping review [16].

### Population

This review focuses on undergraduate nursing students enrolled in an undergraduate nursing degree program.

### Concept

The core concept of this scoping review focuses on the skills evaluation model of undergraduate nursing students. The concept of a skills evaluation model is defined as a structured and theory-informed approach used to assess the progressive development of clinical skills among undergraduate nursing students. This is grounded in Benner’s Novice to Expert theory, in which skill evaluation is viewed as a developmental process through which learners advance from novice to competent practitioners via guided practice, feedback, and reflection [19]. This concept encompasses the frameworks, methods, and tools employed to evaluate and support the acquisition of nursing competence.

### Context

The context includes any undergraduate nursing students enrolled in undergraduate nursing degree programs, inclusive of 3 or 4 years in length, which have clinical skills testing in their program curricula, course evaluation, or as an educational requirement. The scoping review will not focus on accelerated, second-entry, and diploma nursing programs. In addition, registered practical nurse programs will be excluded to ensure the consistency of the scoping review focusing on undergraduate nursing degree programs. This distinction will ensure the findings are pertinent to undergraduate nursing curricula and competencies as the complexity of skills tested might vary between such programs.

### Types of Sources

The scoping review will include any type of studies with quantitative, qualitative, or mixed method designs. Additionally, nonempirical research, such as editorials, opinion papers, gray literature, and reviews will be included. Finally, professional nursing organizations, such as the College of Nurses of Ontario (CNO), the Registered Nurses’ Association of Ontario (RNAO), and the Canadian Association of Schools of Nursing (CASN), will be accessed for any pertinent documents or information on the topic.

### Search Strategy

The scoping review will query the following databases for sources: SCOPUS, CINAHL, Medline, PsycInfo, ProQuest Central, and ProQuest Dissertations and Theses. Moreover, key nursing professional organization websites such as RNAO, CASN, and CNO will also be used. A detailed search syntax for CINAHL has been initiated (see Multimedia Appendix 1). In consultation with a research librarian, the search syntax was developed. This initial search syntax will be modified accordingly to the respective criteria of each identified database and will use standard Boolean operators. This comprehensive search strategy will ensure the breadth of existing literature is captured for this scoping review.

### Study Selection

EndNote software will be used to organize and manage all retrieved sources. Duplicates among the compiled sources will be identified and removed [20]. Subsequently, a preliminary screening will be conducted to enhance the source selection process, which will be followed by independent screening of sources by two reviewers according to titles and abstracts to ensure they meet the review’s inclusion criteria. Sources considered pertinent to the review’s central question will be retrieved and all details will be documented in the JBI’s System for the Unified Management, Assessment, and Review of Information (SUMARI). This is to facilitate a systematic method for assessing, analyzing, and synthesizing the results [21]. Two independent reviewers will analyze the full texts of the retrieved sources to verify the eligibility of each study according to the predetermined inclusion criteria. The justification for omitting sources will be recorded in the PRISMA flow diagram [22]. If any conflicts arise between the reviewers regarding a source’s eligibility, solutions will be pursued through discussions with the third reviewer, the principal investigator. The complete search and selection procedures will be documented in the final scoping review and illustrated within a PRISMA-ScR flow diagram to guarantee transparency and reproducibility of the review [22].

### Data Extraction

All included sources will undergo a data extraction process carried out by two independent reviewers, using a tool specifically developed to gather information relevant to the primary research question of the scoping review. Where applicable, extracted data will capture pertinent information regarding the study’s participants, context, and methods, along with key findings crucial to the review inquiry. The data extraction tool may undergo refinement to align more closely with new findings and all changes will be documented accordingly. In instances where disagreements arise between the reviewers, a third reviewer, the principal investigator of this scoping review, will intervene to efficiently reach a consensus and resolve these disputes.

### Results Synthesis and Presentation

The extracted data will be organized according to emerging themes, while also examining study contexts, methods, results, and conclusions, particularly focusing on common evaluation models currently being used by instructors to evaluate clinical skills in undergraduate nursing students. A qualitative content analysis will follow to uncover key themes, patterns, and trends within the data to facilitate a comprehensive understanding of the research inquiry [16]. This entails the translation of results from quantitative research into classifications that can be integrated with qualitative narratives, guaranteeing a comprehensive synthesis of data across various types of sources included in the final review. The process will start with the initial coding of data, methodically grouping the data into categories according to pre-established and emerging themes. Subsequently, we will refine these preliminary codes into broader themes by meticulously identifying and outlining the relationships between them. Our scoping review will assess the prevalence of key concepts, characteristics, and patterns across the included sources. Our objective is to recognize common themes and trends among these elements, offering a comprehensive examination of the data. To uphold rigor and confirm our findings, two researchers will independently conduct the result syntheses, thereby ensuring the strength and validity of our analysis. The concluding summary will integrate the results from various data sources to form a complete overview of the subject that improves the comprehension of the emergent themes. The results from this analysis will be displayed in both table and diagram format to provide a thorough summary of the scope and range of research regarding the methods of clinical skills evaluation used throughout undergraduate nursing education. Furthermore, a narrative summary will be created to incorporate these findings and align the data with the objectives of this review.

### Ethical Considerations

 This study is a scoping review of the literature and does not involve any human or animal participants. Therefore, research ethics approval was not sought and is not required for this study.

## Results

**Figure 1.**
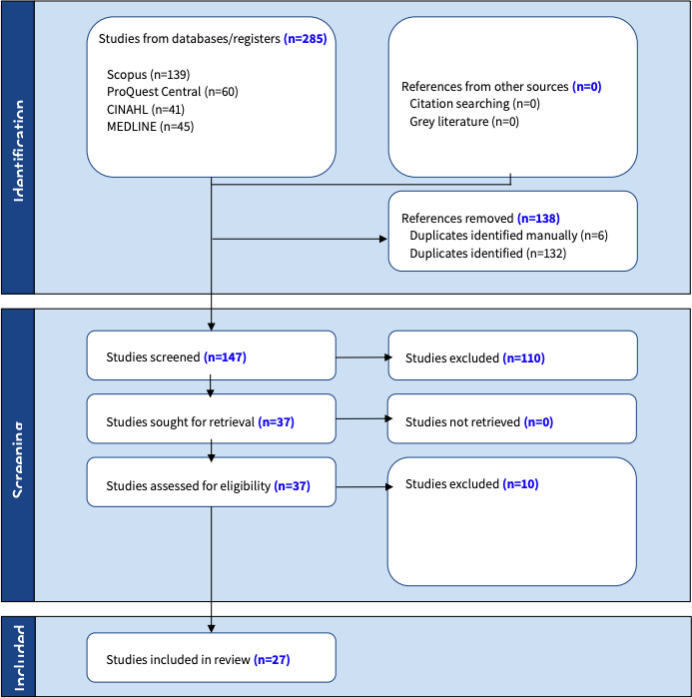
PRISMA (Preferred Reporting Items for Systematic Reviews and Meta-Analyses Extension for Scoping Reviews) flow diagram.

Funding for the project was secured in June 2025. The scoping review is expected to commence in July 2025, with literature searches and the selection of relevant studies continuing throughout Fall 2025 (Figure 1). Findings are anticipated to be ready for publication submission by Winter 2026.

## Discussion

### Anticipated Findings

This scoping review will provide insights for nursing instructors teaching at postsecondary institutions who are involved in developing, revising, and improving undergraduate nursing curricula, particularly in the area of clinical practice preparation and student readiness evaluation. The results will help identify best practices related to the evaluation of undergraduate nursing students’ clinical skills and inform the development of a more inclusive and accurate method of assessment, promoting positive student learning experiences and decreasing academic stress and test-taking anxiety. Furthermore, the findings can highlight gaps in current literature and identify priority areas for research within undergraduate nursing research and education.

Throughout this scoping review, we have recognized two main limitations that may affect the range and depth of our findings. First, due to constraints on translating resources, there is potentially language bias as only English sources will be considered. This limitation may lead to the omission of pertinent studies that have been conducted in other languages, which may provide additional insights or varied perspectives regarding undergraduate nursing education across different geographical regions. However, this scenario is unlikely since the only anticipated omissions would be French-language sources, and given the Canadian context, most literature is expected to be either bilingual or primarily available in English. Second, our review may also be subject to publication bias as studies yielding negative or null results are less likely to be published than those with statistically or clinically significant results.

### Conclusion

Psychomotor skills are an essential component to undergraduate nursing education. However, national guidelines do not currently exist to guide nursing programs and educators on the best way to teach and evaluate nursing students to ensure competent and safe practice within the clinical setting [23,24]. This scoping review aims to provide insights that inform future nursing education by identifying gaps and emerging trends in current clinical skills testing models. By synthesizing evidence from the past decade, the review can highlight current practices and inform future innovative clinical skills testing models that effectively accommodate the needs of a growing nursing student population. How students perceive learning psychomotor skills can offer valuable insight for faculty members into how they can create laboratory environments that support effective student-centered learning and encourage active student involvement throughout the teaching process [25,26].

High-stakes evaluation methods require further exploration of the instruments, tools, or approaches used by educators based on validity, reliability, and objectivity. Combinations of assessment methods should also be considered by faculty to minimize instructor bias and the effect of ‘‘outlier’’ evaluations [27,28]. Using carefully designed testing methods with valid and reliable instruments for the evaluation of competency is the bridge to ensuring the delivery of quality health care and patient safety across all disciplines of professional nursing practice.

The findings of this scoping review will serve as a foundation for revamping existing clinical skills assessment frameworks, ensuring they remain practical and scalable. Specifically, the review can inform educators and administrators in designing future assessment methods that improve the student experience, reduce anxiety associated with clinical evaluations, enhance learning outcomes, and better prepare students for real-world clinical demands. Ultimately, the insights gained will contribute to developing a robust clinical skills testing model tailored to contemporary educational challenges.

## Supplementary material

10.2196/79805 Multimedia Appendix 1 Search strategy.

10.2196/79805 Peer Review Report 1 Peer review report by Toronto Metropolitan University Teaching and Learning Grant Committee, Centre for Excellence in Learning and Teaching, Toronto Metropolitan University.

## Abbreviations

CASN: Canadian Association of Schools of Nursing
CNO: College of Nurses of Ontario
JBI: Joanna Briggs Institute
PRISMA-P: Preferred Reporting Items for Systematic Reviews and Meta-Analysis Protocols extension
PRISMA-ScR: Preferred Reporting Items for Systematic Reviews and Meta-Analyses Extension for Scoping Reviews
RNAO: Registered Nurses’ Association of Ontario
SUMARI: System for the Unified Management, Assessment, and Review of Information
