# Supplementary material for: Evaluation Models for Undergraduate Nursing Clinical Skills: A Scoping Review Protocol
Source: JMIR Res Protoc. 2026 Mar 13;15:e79805. doi: 10.2196/79805 (PMC12986783; doi:10.2196/79805)
Supplement: Multimedia Appendix 1 [file resprot-v15-e79805-s001.docx]

**Appendix I: Search Strategy**

| **CINAHL**  Search conducted: June 23, 2025 | | |
| --- | --- | --- |
| **Search** | **Query** | **Records retrieved** |
| #1 | "clinical skills test" OR "skill pass-offs" OR "clinical skills assessment" OR “clinical skills evaluation” OR "objective structured clinical examination" OR "objective structured clinical assessment" OR "skills checkoff" OR “standardized skills test” OR AB "clinical skills test" OR "skill pass-offs" OR "clinical skills assessment" OR “clinical skills evaluation” OR "objective structured clinical examination" OR "objective structured clinical assessment" OR "skills checkoff" OR “standardized skills test” | 1,184 |
| #2 | "undergraduate nursing education" OR "undergraduate nursing students" OR "Bachelors in Nursing" OR “undergraduate nursing program” OR AB "undergraduate nursing education" OR "undergraduate nursing students" OR "Bachelors in Nursing" OR “undergraduate nursing program” | 4,658 |
| #3 | #1 AND #2 | 36 |
| Limited to English, >2015 | |  |
