# Supplementary material for: Evaluation Models for Undergraduate Nursing Clinical Skills: A Scoping Review Protocol
Source: JMIR Res Protoc. 2026 Mar 13;15:e79805. doi: 10.2196/79805 (PMC12986783; doi:10.2196/79805)
Supplement: Peer Review Report 1 [file resprot-v15-e79805-s002.pdf]

July 15, 2025

Toronto Metropolitan University

Centre for Excellence in Learning & Teaching

Dear Professor Kateryna Metersky,

This document contains the feedback and scores submitted by the two adjudicators assigned to review your application.

Thank you again for your contributions.

Sincerely,

Esther Ignagni

Executive Director

Centre for Excellence in Learning and Teaching

Daphne Cockwell Centre for Health Sciences (DCC-715)
288 Church Street
Toronto, ON, Canada M5B 1Z5
torontomu.ca/teachingcentre
teachingcentre@torontomu.ca

**Metersky, Kateryna**<br><br>**An Environmental Scan and Exploration of Best Practices to Develop a New Skills Testing Model for Undergraduate Nursing Students**<br><br>**Daphne Cockwell School of Nursing**
**Adjudicator #1**
**PART ONE: Project Rationale**<br>1.1). The development or innovation and the goal, opportunity, challenges, and connections to supporting EDIA+ D to be addressed is: 4/5<br><br>1.2). The ways in which existing pedagogical scholarship and any current work at TMU informs the proposed project is: 4/5<br><br>**Comments**: The proposal effectively outlined the lack of flexibility / accommodation with the current Nursing assessment model and in order to equitably support varied routes to academic success, as well as reduce undue anxiety for students, this project is very much needed. Add to that the government mandates an increase in enrollment, the project is very timely. While the proposal does mention the TMU Nursing handbook and TMU's Policy No. 159, it lacks reference to more robust scholarship
**PART TWO: Priority Alignment**<br>2.1). The alignment between the proposed project and the focus area(s) is:5/5<br><br>**Comments**:Highly relevant to First Year Experience and Mental Health (although authors did not name the mental health focus area)<br>Relevant to Experiential Learning focus.
**PART THREE: Activities Undertaken to Support the Development/Innovation**<br><br>3.1) The activities that will be undertaken to support the development or innovation are: 4/5<br><br>3.2) Within the design of the activities, the principles of decolonization, equity, diversity, and inclusion are: 4/5<br><br>3.3) Timeline Form: 4/5<br><br>**Comments**:While the team will be drawing from environmental scan and literature findings to "*ensure the new model is accessible and culturally responsive*" it may be prudent to add (a) the identification and consultation with EDIA and accommodation experts at TMU as an activity for model validation.<br>I have a bit of concern with allotting 20 hours of undergraduate student time to

prepare a manuscript for publication. Uncertain if this is feasible.
**PART FOUR: Impact on Teaching and Student Learning**<br><br>4.1). The intended impacts of the proposed project on improving teaching and student learning and its link back to the goals, opportunities and/or challenges, and context you described above are: 3.5/5<br><br>4.2) How the impact of the innovation will be measured or assessed are: 3/5<br><br>4.3) How the work will benefit others within and outside of their discipline is: 4/5<br><br>4.4) How will the findings be shared locally and beyond is: 4.5/5<br><br>4.5) How will these impacts be sustained beyond the funding period for the project is: 4/5<br><br>**Comments:** Plan for assessment is lacking - consists of new testing model adoption and implementation. Impact for TMU students is clear, along with GB and Centennial students, and beyond.
**PART FIVE: Students as Partners**<br><br>5.1 The ways in which students and their perspectives will be engaged through the proposed project are: 5/5<br><br>**Comments:** Student input via focus groups and hired to execute the project.
Applications includes, budget justification, citations, timeline and signature form: YES
Overall feedback:<br>The work outlined in this proposal is very important, timely, and very much needed. Perhaps a second associated project is warranted prior to full implementation of the new model – testing the new model with students/instructors. Perhaps a testing period during which two parallel assessment models are utilized in the curriculum.

**Metersky, Kateryna**<br><br>**An Environmental Scan and Exploration of Best Practices to Develop a New Skills**<br>**Testing Model for Undergraduate Nursing Students**<br><br>**Daphne Cockwell School of Nursing**
**Adjudicator #2**

PART ONE: Project Rationale

1). The development or innovation and the goal, opportunity, challenges, and connections to supporting EDIA+ D to be addressed is: 4/5

1.2). The ways in which existing pedagogical scholarship and any current work at TMU informs the proposed project is: ⅗

Comments: Would have liked to see more tangible evidence of current problematic (i.e. course evaluations) and comments from faculty who supervise. The citation included touches on a variety of stressors for Nursing students, only one of which is skills testing and that stressor is most evident in 2nd year students in a very small sample size.

PART TWO: Priority Alignment

2). The alignment between the proposed project and the focus area(s) is: 3/5

Comments: Stronger connection to First Year Experience over Experiential learning (as per specifications)

PART THREE: Activities Undertaken to Support the Development/Innovation

3.1 The activities that will be undertaken to support the development or innovation are: 3/5

3.2 Within the design of the activities, the principles of decolonization, equity, diversity, and inclusion are: 2/5

3.3 Timeline Form: ⅗

Comments:The scoping review is very likely to be unnecessary - if anything, an umbrella review of existing review articles will suffice (i.e. there are 100+ scoping studies about nursing skills indexed in CINAHL with the most recent one from January 2025); the environmental scan is likely to be more informative, but could likely be undertaken more efficiently through engaging with the CASN framework. Time saved on these aspects could be used to kickstart the REB and recruitment phases.

PART FOUR: Impact on Teaching and Student Learning

4.1) The intended impacts of the proposed project on improving teaching and student learning and its link back to the goals, opportunities and/or challenges, and context you described above are: 4/5

4.2) How the impact of the innovation will be measured or assessed are: 3/5

4.3) How the work will benefit others within and outside of their discipline is: 3/5

4.4) How will the findings be shared locally and beyond is: 4/5

4.5) How will these impacts be sustained beyond the funding period for the project is: 3/5<br><br>**Comments:** Impacts are mainly focussed within the discipline. Sustainability is only defined as a curricular update with no indication of ongoing evaluation.
**PART FIVE: Students as Partners**<br><br>5.1 The ways in which students and their perspectives will be engaged through the proposed project are: 4/5<br><br>**Comments:** None.
Applications includes, budget justification, citations, timeline and signature form: YES
Overall feedback:<br><br>None
